# Detection of mutations in *KLHL3* and *CUL3* in families with FHHt (familial hyperkalaemic hypertension or Gordon's syndrome)

**DOI:** 10.1042/CS20130326

**Published:** 2014-02-03

**Authors:** Mark Glover, James S. Ware, Amanda Henry, Martin Wolley, Roddy Walsh, Louise V. Wain, Shengxin Xu, William G. Van’t Hoff, Martin D. Tobin, Ian P. Hall, Stuart Cook, Richard D. Gordon, Michael Stowasser, Kevin M. O’Shaughnessy

**Affiliations:** *Division of Therapeutics and Molecular Medicine, University of Nottingham, Nottingham, U.K.; †NIHR Biomedical Research Unit in Cardiovascular Disease at Royal Brompton and Harefield NHS Foundation Trust and Imperial College London, London, U.K.; ‡National Heart and Lung Institute, Imperial College, London, U.K.; §Endocrine Hypertension Research Centre, University of Queensland School of Medicine, Brisbane, Australia; ¶Genetic Epidemiology Group, University of Leicester, Leicester, U.K.; ∥Paediatric Nephrology Department, Great Ormond Street Hospital for Children, London, U.K.; **Cardiovascular and Metabolic Disorders Program, Duke-National University of Singapore, Singapore; ††National Heart Centre Singapore, Singapore; ‡‡Clinical Pharmacology Unit, University of Cambridge, Cambridge, U.K.

**Keywords:** diuretic, Gordon's syndrome, hypertension, hyperkalaemia, pseudohypoaldosteronism, thiazide, CUL3, cullin 3, FHHt, familial hyperkalaemic hypertension, GAN, gigaxonin, IBD, identity by descent, KLHL3, kelch-like family member 3, NCC, Na^+^–Cl^−^ co-transporter, NGS, next-generation sequencing, SLC, solute carrier, SNP, single nucleotide polymorphism, SPAK, STE20/SPS1-related proline/alanine-rich kinase, STE20, sterile 20, WNK, With No lysine (=K)

## Abstract

The study of families with rare inherited forms of hypo- and hyper-tension has been one of the most successful strategies to probe the molecular pathophysiology of blood pressure control and has revealed dysregulation of distal nephron Na^+^ reabsorption to be a common mechanism. FHHt (familial hyperkalaemic hypertension; also known as Gordon's syndrome) is a salt-dependent form of hypertension caused by mutations in the regulators of the thiazide-sensitive Na^+^–Cl^−^ co-transporter NCC [also known as SLC12A3 (solute carrier family 12 member 3)] and is effectively treated by thiazide diuretics and/or dietary salt restriction. Variation in at least four genes can cause FHHt, including *WNK1* [With No lysine (=K) 1] and *WNK4*, *KLHL3* (kelch-like family member 3), and *CUL3* (cullin 3). In the present study we have identified novel disease-causing variants in *CUL3* and *KLHL3* segregating in 63% of the pedigrees with previously unexplained FHHt, confirming the importance of these recently described FHHt genes. We have demonstrated conclusively, in two unrelated affected individuals, that rare intronic variants in *CUL3* cause the skipping of exon 9 as has been proposed previously. *KLHL3* variants all occur in kelch-repeat domains and so probably disrupt WNK complex binding. We have found no evidence of any plausible disease-causing variants within *SLC4A8* (an alternative thiazide-sensitive sodium transporter) in this population. The results of the present study support the existing evidence that the *CUL3* and *KLHL3* gene products are physiologically important regulators of thiazide-sensitive distal nephron NaCl reabsorption, and hence potentially interesting novel anti-hypertensive drug targets. As a third of our non-*WNK* FHHt families do not have plausible *CUL3* or *KLHL3* variants, there are probably additional, as yet undiscovered, regulators of the thiazide-sensitive pathways.

## Abstract 2

The present study has found new mutations in the *CUL3* and *KLHL3* genes of patients with Gordon's syndrome. *CUL3* mutations were shown to cause a defect in the splicing of exon 9. One-third of families with Gordon's syndrome remain without a genetic diagnosis.

## INTRODUCTION

Hypertension is estimated to contribute 3.5-fold more to the total global disease burden of cardiovascular disease than smoking and 1.6-fold that of hypercholesterolaemia. Worldwide, 20% of deaths in men, 24% of deaths in women, 62% of strokes and 49% of heart disease are attributable to blood pressure [1–3]. The current limitations in anti-hypertensive therapeutics are perhaps not surprising since for most affected individuals the molecular mechanisms driving their hypertension remain undefined.

Although rare, Mendelian forms of hypo- and hyper-tension represent experiments of Nature that have informed our understanding of the physiology of the distal nephron. Remarkably, given the variety of physiological systems that affect arterial pressure, all of these Mendelian syndromes for which the molecular mechanism is understood converge around a common theme: distal nephron sodium wasting in hypotensive syndromes and excessive sodium reabsorption in hypertensive conditions [4].

Although the amiloride-sensitive ENaC (epithelial sodium channel) has classically dominated research interests, NaCl reabsorption via the thiazide-sensitive Na^+^–Cl^−^ co-transporter NCC [also known as SLC12A3 (solute carrier family 12 member 3)] is at least as important [5]. Thiazide diuretics are potent anti-hypertensive agents [6] and mimic the effects of loss-of-function mutations of NCC observed in the hypotensive monogenic syndrome of Gitelman [7]. Moreover, the heritable condition of FHHt (familial hyperkalaemic hypertension) results from increased sodium reabsorption via NCC and is effectively ameliorated by thiazide diuretics and/or dietary sodium restriction [8].

FHHt is a salt-sensitive hypertension characterized by hyperkalaemic acidosis and exquisite sensitivity to low-dose thiazide diuretics [8,9]. As in Liddle's syndrome [10], significant inter- and intra-pedigree phenotypic variation is observed clinically [11]. Causative variants have been identified in *WNK1* [With No lysine (=K) 1] and *WNK4*, *KLHL3* (kelch-like family member 3), and *CUL3* (cullin 3) [12–15], but not within the NCC itself [16]. Variants are inherited in an autosomal dominant or recessive manner depending on the gene involved and can also occur *de novo* [8,14].

The current model for the regulation of NCC is complex and involves a scaffold of at least 12 interacting proteins centred on a WNK signalling cascade, with intermediary STE20 (sterile 20) kinases [SPAK (STE20/SPS1-related proline/alanine-rich kinase) and OSR1 (oxidative stress-responsive kinase-1)] activated by WNKs which in turn activate NCC [17–19]. CUL3 and KLHL3 are both components of the cullin/Ring E3 ligase ubiquitination pathway and at least some variants of *KLHL3* appear to affect NCC via the control of WNK1 ubiquitination [15,20].

We have identified previously three FHHt pedigrees carrying *WNK4* mutations (D564H, E562K and Q565E) [21], but none carrying *WNK1* mutations. To assess whether our remaining pedigrees with FHHt and without *WNK1/4* mutations had either *CUL3* or *KLHL3* mutations, we undertook NGS (next-generation sequencing) of these genes and also screened an alternative thiazide-sensitive sodium transporter (*SLC4A8*) hypothesized to be an additional candidate [14].

## MATERIALS AND METHODS

### Study population

The present study was carried out in accordance with the Declaration of Helsinki (2013) of the World Medical Association. Study participants with an FHHt phenotype were identified through tertiary specialist hypertension clinics in the U.K. and Australia. Diagnosis of FHHt was confirmed by the authors. All affected patients were Caucasian and shared a phenotype of persistent hyperkalaemia (plasma potassium >5.0 mmol/l in blood collected without stasis) and hypertension (>140/90 mmHg for adults) following exclusion of the relevant co-morbidities and pharmacotherapies. Detailed phenotypes of the affected individuals are given in Supplementary Figure S1 (at http://www.clinsci.org/cs/126/cs1260721add.htm). All non-affected individuals demonstrated plasma potassium <5 mM/l. The disparity in ages prevented comparison of age-related blood pressure between affected and non-affected individuals. DNA was extracted using a standard method from venous blood acquired following informed consent (Princess Alexandra Hospital Human Research Ethics Committee ID EC00167 in Australia and National Research Ethics Committee reference 12/EM/0317 in the U.K.).

### DNA analysis

*CUL3*, *KLHL3* and *SLC4A8* genes were sequenced in the affected proband of each family using NGS. PCR amplicons covering all coding exons and exon/intron boundaries were prepared from genomic DNA (Fluidigm Access Array™; the amplicons used are listed in Supplementary Table S1 at http://www.clinsci.org/cs/126/cs1260721add.htm) and sequenced on the Illumina HiSeq platform. Reads were aligned to the human reference sequence hg19 using the Burrows–Wheeler Aligner, and the Genome Analysis Toolkit was used for base recalibration, local realignment and variant calling, following published best practice guidelines, and as described previously [22]. Variants were filtered for rarity and protein consequence: variants altering the protein-coding sequence [missense and non-sense SNPs (single nucleotide polymorphisms), insertions or deletions, or intronic variants at the exon/intron boundary] that were absent from public databases [dbSNP, 1000 Genomes and the NHLBI ESP (National Heart, Lung, and Blood Institute Exome Sequencing Project) Exome Variant Server] were considered candidates. All candidates detected by NGS were confirmed in the proband and assessed for segregation in the pedigree using Sanger sequencing. Variants are reported using Human Genome Variation Society standard nomenclature (http://www.hgvs.org/mutnomen/). The reference sequences used for each gene and protein are listed in Supplementary Table S2 (at http://www.clinsci.org/cs/126/cs1260721add.htm).

### RNA studies

The functional effects of putative splice variants were confirmed using RNA studies. Peripheral blood mononuclear cell RNA was isolated from 5 ml of whole blood using a PAXgene blood RNA kit (Qiagen) according to the manufacturer's instructions. The RNA was then transcribed using a Promega AMV reverse transcriptase kit (catalogue number A3500) according to manufacturer's instructions using either random primers (RT1) or a *CUL3*-specific primer (5′-TTATGCTACATATGTGTATAC-TTTGC-3′; RT2). The resulting cDNA was then PCR-amplified using exon-specific primers to amplify exons 8–10 of the *CUL3* transcript (forward, 5′-TCAACCTCAACTCCAGGTCTCC-3′ and reverse, 5′-TGTTGCCTGAATTCATCCATCG-3′). The PCR products were run on a 2% agarose gel to visualize them, excised, cleaned using a Promega PCR clean-up kit and Sanger-sequenced on a Beckman CEQ 6800 sequencer. The expected PCR product sizes were 338 bp and 167 bp for the exon 8–10 and del9 transcripts respectively.

### Paralogue mapping

For each gene we first identified paralogues using pre-defined Ensembl protein families (http://www.ensembl.org; release 70), and constructed a multiple sequence alignment using M-Coffee [23]. Reported Mendelian disease-causing variants (non-synonymous SNPs causing a single non-terminal amino acid change) in paralogues of the FHHt genes were identified using the Human Gene Mutation Database Professional version (http://www.hgmd.cf.ac.uk; release 2012.3), and mapped to the equivalent residue of the FHHt gene in the multiple sequence alignment.

### Exon-directed array and identity by descent analysis

Representative affected individuals in pedigrees 6, 7 and 8 were genotyped using the Illumina Infinium HumanExome BeadChip array. Pair-wise IBD (identity by descent) analysis was undertaken using PLINK version 1.0.7 [24] on the basis of a subset of 27402 informative autosomal SNPs with a minor allele frequency >5%. A proportion of IBD (PI_HAT) <0.05 was considered to indicate no excess of sharing (i.e. unrelated individuals).

## RESULTS

Genetic analysis of 25 affected individuals from 16 families with FHHt who had already been screened and found negative for *WNK1/4* mutations was performed. A total of 95% of the targeted bases were sequenced adequately for variant calling. The sequencing depth and coverage achieved by gene and exon are shown in Supplementary Figure S2 (at http://www.clinsci.org/cs/126/cs1260721add.htm).

Affected individuals (*n*=16) from ten of these 16 families were found to have *CUL3* or *KLHL3* variants not reported in the general population (Table 1 and Supplementary Figure S3 at http://www.clinsci.org/cs/126/cs1260721add.htm). We found no evidence of rare variants in *SLC4A8* which segregated with disease phenotype.

**Table 1:** *CUL3* and *KLHL3* variants segregating with the FHHt phenotype in each pedigree The zygosity of affected individuals within each pedigree for the causative variant is shown. Conservation describes the KLHL3 amino acid residues conserved across species expressed as the proportion of species sharing the same reference allele in primates (P), mammals (M) and vertebrates (V) (Ensemble KLHL3 paralogues; available at http://www.ensembl.org/Homo_sapiens/Gene/Compara_Ortholog?g=ENSG00000146021;r=5:136953189-137071779). The country of origin of each pedigree is also shown. Variants are described according to Human Genome Variation Society (HGVS) standard nomenclature using the reference sequences listed in Supplementary Table S2 (at http://www.clinsci.org/cs/126/cs1260721add.htm). *Previously undescribed variants; **previously undescribed genotype.

Pedigree	Gene	Genomic DNA position	HGVS coding DNA position	Zygosity	rs_identity	Protein effect	Conservation	Country of origin
1*	*CUL3*	Chr2:225368368	c.1377+1G>T	Heterozygous	–	Exon 9/intron 9 splicing	–	U.K.
2	*CUL3*	Chr2:225368368	c.1377+1G>C	Heterozygous	rs199469660	Exon 9/intron 9 splicing	–	Australia
3*	*CUL3*	Chr2:225368551	c.1207–12T>A	Heterozygous	–	Exon 9/intron 8 splicing	–	Australia
4	*CUL3*	Chr2:225368540	c.1207–1G>A	Heterozygous	rs199469654	Exon 9/intron 8 splicing	–	U.K.
5**	*KLHL3*	Chr5:136964078	c.1499G>T	Homozygous	–	G500V	P (9/9), M (37/37), V (49/50)	U.K.
6/7/8	*KLHL3*	Chr5:136974701	c.1160T>C	Heterozygous	rs199469630	L387P	P (9/9), M 33/33), V (46/46)	Australia
9	*KLHL3*	Chr5:136975551	c.1019C>T	Heterozygous	rs199469628	A340V	P (9/9), M 30/31), V (43/44)	Australia
10	*KLHL3*	Chr5:136964097	c.1480G>A	Heterozygous	rs199469633	A494T	P (9/9), M 36/36),	U.K.

As shown in Table 1 and Supplementary Figure S2, affected individuals from eight pedigrees carried variants that have been associated previously with FHHt, two in *CUL3* and six in *KLHL3*. Affected individuals from two pedigrees carried variants unreported previously in *CUL3* (c.1207-12T>A and c.1377+1G>T). In addition an affected individual from pedigree 5 was homozygous for a previously reported heterozygous *KLHL3* variant (c.1499G>T; p.G500V) [15]. In keeping with previous observations, *CUL3* mutations were intronic and probably affected splicing of exon 9, whereas *KLHL3* mutations were non-synonymous exonic SNPs (Supplementary Figures S4 and S5 at http://www.clinsci.org/cs/126/cs1260721add.htm).

**Figure 1:**
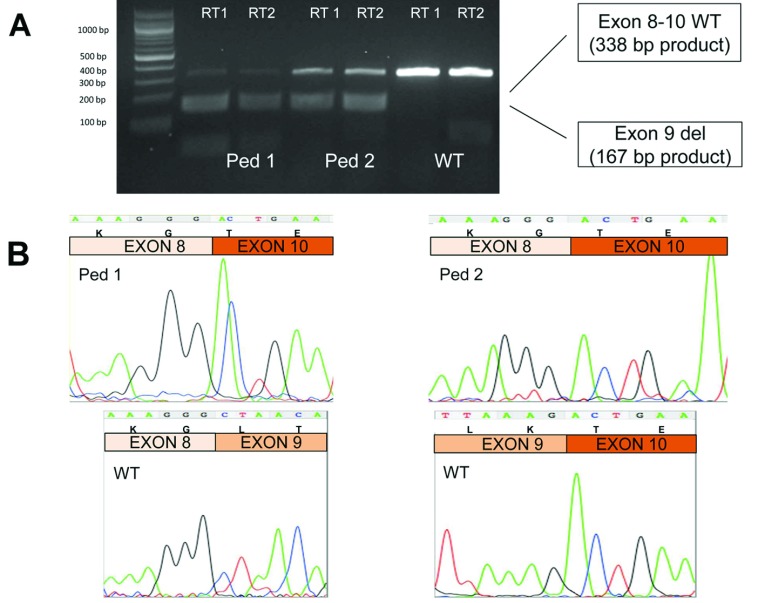
Demonstration that the *CUL3* variants result in splice variation leading to a loss of exon 9 in affected individuals from pedigree 1 (Ped1) and pedigree 2 (Ped2) The affected individuals sequenced are highlighted by * in Supplementary Figure S3 (at http://www.clinsci.org/cs/126/cs1260721add.htm). (**A**) Reverse transcription–PCR of *CUL3* from peripheral blood mononuclear cells demonstrated an additional (smaller) cDNA band only in the affected individuals. The size of the smaller band was consistent with a deficiency of exon 9 (difference in band size=171 bp). PCR primers RT1 (random primers) and RT2 (a *CUL3*-specific primer) are detailed in the Materials and methods section. The molecular size is given on the left-hand side in bp. (**B**) Sanger sequencing of *CUL3* cDNA from the smaller 167 bp band confirmed that exon 9 is skipped in individuals from both pedigrees. Sequence excerpts from the larger 338 bp band are shown for the wild-type (WT) individual for comparison, demonstrating the wild-type exon boundaries. Sequencing chromatograms are shown together with the DNA sequence and amino acid codons above.

An affected individual from pedigree 1 had an alternative G>T variant at the same position in *CUL3* as one from pedigree 2 (c.1377+1G>C; the original proband reported by Gordon et al. [9]). Both had a severe hyperkalaemic phenotype apparent during childhood despite coming from different families and living on opposite sides of the globe. Although variants surrounding this exon 9/intron 9 acceptor splice site have been predicted to affect splicing of exon 9 [14], the present study has provided the first evidence of this effect in FHHt patients. Specifically, RNA from peripheral blood monocytes of the index case in pedigrees 1 and 2 contains exon 9-deficient transcripts from the mutated *CUL3* allele (Figure 1).

Pedigrees 6, 7 and 8 all carry the same KLHL3 p.L387P mutation that segregates completely with an FHHt phenotype, raising the question whether these families have a common founder. IBD analysis (Illumina Infinium HumanExome BeadChip) revealed that these pedigrees were no more related than by chance (PI_HAT=0.0440), indicating that the mutation has probably arisen independently in each lineage. Although the *KLHL3* R528H mutation has also been reported in three pedigrees [15], it was not established whether they shared a common founder. Hence in our pedigrees, KLHL3 p.L378P is the most commonly identified FHHt-causing *KLHL3* mutation with robust evidence of independent founder mutations.

To assess the pathogenicity of the KLHL3 variants associated with FHHt, we used a Paralogue Annotation approach [25]. KLHL3 is one of a family of evolutionarily related cytoskeletal BTB/kelch repeat proteins, variation in several of which cause Mendelian disease. Using multiple sequence alignment to identify structurally and functionally equivalent residues across the protein family, we observed that one of the KLHL3 variants reported previously to be associated with FHHt [14,15] (KLHL3 p.R384W) co-locates with a reported disease-causing variant in another member of the protein family KBTBD13 p.R248S {where KBTBD13 is kelch repeat and BTB [BR-C (Broad Complex), ttk (tramtrack) and bab (bric a brac)] (POZ) domain-containing 13}, which is associated with nemalin myopathy [26]. This suggests that the variants lie at a functionally important site conserved across the protein family that is intolerant of sequence variation. Similarly, two of the KLHL3 FHHt variants in our patients (L387P and A494T) are very close to the location of known disease-causing variants in GAN (gigaxonin) [27], suggesting that these too are probably functionally important sites. GAN p.G368 and p.G474 (at which substitutions are associated with giant axonal neuropathy [27]) are equivalent to KLHL3 p.G388 and p.G496, and are adjacent to rare variants found in our FHHt pedigrees.

## DISCUSSION

In the present study we have identified disease-causing variants in *CUL3* and *KLHL3* in 63% of our pedigrees with FHHt who had been screened and found to be negative for *WNK1/4* mutations, confirming recent reports of association between *CUL3* and *KLHL3* variants and FHHt [14,15]. In the case of *CUL3* mutation at position c.1377+1 we report a second variant allele associated with a similar thiazide-responsive FHHt phenotype, strengthening further the case for a functional role of aberrant CUL3 function on sodium reabsorption in the distal nephron. We have also demonstrated that the predicted exon 9 splicing effect produced by c.1377+1G>T and c.1377+1G>A is, in fact, observed.

We have found that KLHL3 p.L387P associated with FHHt in three unrelated pedigrees, making this the most commonly occurring single FHHt mutation not only within our FHHt consortium, which includes three FHHt pedigrees carrying different WNK4 mutations (D564H, E562K and Q565E) [21], but also among all *KLHL3* mutations reported to date [14,15]. That *KLHL3* variants in our pedigrees are restricted to kelch repeats, and that other FHHt-associated *KLHL3* variants cluster in these domains provides further support for disruption of WNK complex binding as reported previously [20].

Accepting the limitations of bioinformatics tools to predict pathogenicity, we did not find evidence of probable disease-causing variants within an alternative thiazide-sensitive sodium bicarbonate exchanger, *SLC4A8*, hypothesized as an alternative genetic candidate for FHHt [14]. A third of our pedigrees with non-*WNK* FHHt therefore remain without a genetic diagnosis, which is somewhat greater than that reported in other pedigree collections [14,15]. This highlights the genetic heterogeneity of the FHHt phenotype and the likelihood that additional, as yet undiscovered, regulators of thiazide-sensitive pathways exist. It is also worth emphasizing that we set out to identify *KLHL3* and *CUL3* variants in subjects with a clinical diagnosis of FHHt on the basis of measurements routinely recorded in the clinic. Similar data are recorded for unaffected relatives, but because of the large disparity in ages it is often impossible to provide a comparison of age-related blood pressure between affected and non-affected individuals. Nevertheless, all non-affected individuals were normokalaemic with a plasma potassium <5 mmol/l, and we are confident that we have correctly assigned affected compared with non-affected status within our pedigrees.

Further detailed laboratory and clinical studies are required to establish whether the effects of the reported heterogeneity of variant *KLHL3* on WNK1 immunoprecipitation and ubiquitination translate into differential effects on thiazide-sensitive distal nephron sodium trafficking and phenotype within FHHt [20]. For instance, do patients with KLHL3 A340V and A494T Gordon's syndrome have the same CUL3/KLHL3/WNK/SPAK/NCC pathway abnormalities as those with KLHL3 L387P?

In conclusion we have identified disease-causing variants in *CUL3* and *KLHL3* in patients with FHHt screened previously and found to be negative for *WNK1* and *WNK4* mutations, but did not find evidence of such variants in the alternative candidate *SLC4A8*. Approximately one-third of our non-WNK patients with FHHt remain without a molecular diagnosis raising the possibility that there may be additional regulators of thiazide-sensitive distal nephron sodium trafficking which remain to be discovered.

## CLINICAL PERSPECTIVES

• The present study was performed to acertain whether pedigress with FHHt, but without mutation in *WNK1/WNK4*, contained mutation in *CUL3*, *KLHL3* or *SLC4A8*.
• The present study confirms recent findings of *CUL3* and *KLHL3* mutations in FHHt and identifies novel disease-causing variants. This strengthens the argument that these gene products are physiologically important regulators of distal nephron NaCl reabsorption via thiazide-sensitive pathways, and hence are potentially interesting novel anti-hypertensive drug targets.
• As only 63% of our non-*WNK* FHHt families were found to contain plausible *CUL3* or *KLHL3* variants, there are probably additional, as yet undiscovered, regulators of thiazide-sensitive pathways.
